# STAG2 is a clinically relevant tumor suppressor in pancreatic ductal adenocarcinoma

**DOI:** 10.1186/gm526

**Published:** 2014-01-31

**Authors:** Lisa Evers, Pedro A Perez-Mancera, Elizabeth Lenkiewicz, Nanyun Tang, Daniela Aust, Thomas Knösel, Petra Rümmele, Tara Holley, Michelle Kassner, Meraj Aziz, Ramesh K Ramanathan, Daniel D Von Hoff, Holly Yin, Christian Pilarsky, Michael T Barrett

**Affiliations:** 1 Clinical Translational Research Division, Translational Genomics Research Institute, Scottsdale, AZ 85259, USA; 2 CRUK Cambridge Institute, University of Cambridge, Li Ka Shing Centre, Robinson Way, Cambridge CB2 0RE, UK; 3 Cancer and Cell Biology Division, Translational Genomics Research Institute, Phoenix, AZ 85004, USA; 4 Institute of Pathology, University Hospital Dresden, Fetscherstr, 74, 01307 Dresden, Germany; 5 Institute of Pathology, Ludwig-Maximilians-University (LMU), Thalkirchnerstr. 36, 80337 Munich, Germany; 6 Institute of Pathology, University of Regensburg, Franz-Josef-Strauss-Allee 11, 93053 Regensburg, Germany; 7 Virginia G. Piper Cancer Center, Scottsdale Healthcare, Scottsdale, AZ 85258, USA; 8 Department of Surgery, University Hospital Dresden, Fetscherstr, 74, 01307 Dresden, Germany

## Abstract

**Background:**

Pancreatic ductal adenocarcinoma (PDA) is a highly lethal cancer characterized by complex aberrant genomes. A fundamental goal of current studies is to identify those somatic events arising in the variable landscape of PDA genomes that can be exploited for improved clinical outcomes.

**Methods:**

We used DNA content flow sorting to identify and purify tumor nuclei of PDA samples from 50 patients. The genome of each sorted sample was profiled by oligonucleotide comparative genomic hybridization and targeted resequencing of *STAG2*. Transposon insertions within *STAG2* in a *KRAS*^G12D^-driven genetically engineered mouse model of PDA were screened by RT-PCR. We then used a tissue microarray to survey STAG2 protein expression levels in 344 human PDA tumor samples and adjacent tissues. Univariate Kaplan Meier analysis and multivariate Cox Regression analysis were used to assess the association of STAG2 expression relative to overall survival and response to adjuvant therapy. Finally, RNAi-based assays with PDA cell lines were used to assess the potential therapeutic consequence of STAG2 expression in response to 18 therapeutic agents.

**Results:**

*STAG2* is targeted by somatic aberrations in a subset (4%) of human PDAs. Transposon-mediated disruption of *STAG2* in a *KRAS*^G12D^ genetically engineered mouse model promotes the development of PDA and its progression to metastatic disease. There was a statistically significant loss of STAG2 protein expression in human tumor tissue (Wilcoxon-Rank test) with complete absence of STAG2 staining observed in 15 (4.3%) patients. In univariate Kaplan Meier analysis nearly complete STAG2 positive staining (>95% of nuclei positive) was associated with a median survival benefit of 6.41 months (*P* = 0.031). The survival benefit of adjuvant chemotherapy was only seen in patients with a STAG2 staining of less than 95% (median survival benefit 7.65 months; *P* = 0.028). Multivariate Cox Regression analysis showed that STAG2 is an independent prognostic factor for survival in pancreatic cancer patients. Finally, we show that RNAi-mediated knockdown of STAG2 selectively sensitizes human PDA cell lines to platinum-based therapy.

**Conclusions:**

Based on these iterative findings we propose that STAG2 is a clinically significant tumor suppressor in PDA.

## Background

A genetic hallmark of pancreatic ductal adenocarcinoma (PDA) is the presence of somatic *KRAS* mutations in over 90 to 95% of tumors, the most prevalent being *KRAS*^G12D^[1,2]. A fundamental question remains the identification of somatic aberrations arising in the complex genomic landscape of PDA that drive the progression of *KRAS* mutant neoplastic cells in humans *in vivo*. Furthermore, of significant interest are those selected aberrations that create therapeutic vulnerabilities that can be exploited to advance improved and more personalized care of patients. The *STAG2* gene encodes a subunit of the cohesion complex, which plays an essential role in the proper division and segregation of chromosomes, a process that is essential for the maintenance of genome stability and cell survival [3,4]. Mutations targeting this class of genes have been studied in model systems, but have been detected in a relatively small number of somatic tumors arising in patients *in vivo*[5-7]. Recently, somatic aberrations and loss of STAG2 expression have been reported in a subset of tumors and cell lines, including melanomas, sarcomas, and glioblastomas [3]. Notably, truncating mutations in *STAG2* have been shown to be one of the most common genetic lesions in bladder carcinoma [8]. Functional analysis has shown that loss of STAG2 leads to chromosome missegregation and aneuploidy in human cell lines and may promote a mutator phenotype [3,4]. The development of a *KRAS*^G12D^-driven genetically engineered mouse (GEM) model of PDA has provided a powerful resource for the study of the events that accelerate tumorigenesis and drive tumor progression in the pancreas [9]. Strikingly, *STAG2* was one of the most frequent and significant insertion targets reported in a transposon-mediated screen of the *KRAS*^G12D^ GEM model of PDA. However, mutations in *STAG2* and clinically relevant variations in its protein expression levels have not been reported to date in human PDA samples [3,8]. Thus, the clinical significance of STAG2 expression and its role as a tumor suppressor gene in human PDA remains to be elucidated.

PDA is a highly lethal cancer that is difficult to molecularly characterize at the biopsy level due to complex genomes and heterogeneous cellularity, as cancer cells represent, on average, only 25% of the cells within the tumor [10]. The presence of admixtures of non-neoplastic cells in patient samples can obscure the detection of somatic aberrations, including mutations, homozygous deletions, and breakpoints, in biopsies of interest. Furthermore, clinical samples frequently contain multiple neoplastic populations that cannot be distinguished by morphology-based methods [11,12]. In order to investigate whether *STAG2* is a tumor suppressor in human PDA, we used DNA content-based flow cytometry to sort PDA samples from 50 patients. The genome of each sorted tumor population was then interrogated for somatic mutations and aberrations with oligonucleotide array comparative genomic hybridization (aCGH) and targeted resequencing using our established protocols [13]. In this present study we also sought to confirm the inactivation of *STAG2* in those *KRAS*^G12D^ GEM tumors with transposon insertions within the gene to provide further evidence of a tumor suppressor role in PDA. We then used a tissue microarray (TMA) to survey STAG2 protein expression levels in 344 human PDA tumor samples and adjacent tissues. The clinical annotation available for each tumor represented on the TMA allowed the assessment of STAG2 expression relative to overall survival and responses to adjuvant therapy. Finally, given the role of *STAG2* in maintaining genomic stability, we used RNA interference (RNAi)-based cellular assays with PDA cell lines to assess the potential therapeutic consequence of STAG2 expression in response to a panel of 18 currently used therapeutic agents. Our results provide evidence for a clinically relevant tumor suppressor role for *STAG2* in *KRAS* mutant PDA. These highly iterative findings have implications for the development of personalized approaches for patients with PDA.

## Methods

### Clinical samples

PDA samples were obtained under a Western Institutional Review Board protocol (20040832) for a National Institutes of Health-funded bio-specimen repository (NCI P01 grant CA109552) and two American Association for Cancer Research/Stand up to Cancer (SU2C) sponsored clinical trials, 20206-001 and 2026-003. Additional PDA samples were obtained with approved consent of the Ethics Committee of Basel (252/08, 302/09). All patients in this study gave informed consent for collection and use of all the samples, which were collected in liquid nitrogen and stored at -80°C. All tumor samples were histopathologically evaluated prior to genomic analysis. All research conformed to the Helsinki Declaration [14].

### Flow cytometry

Biopsies were minced in the presence of NST buffer and DAPI according to published protocols [11,15,16]. Nuclei were disaggregated then filtered through a 40 μm mesh prior to flow sorting with an Influx cytometer (Becton-Dickinson, San Jose, CA, USA) with ultraviolet excitation and DAPI emission collected at >450 nm. DNA content and cell cycle were analyzed using the software program MultiCycle (Phoenix Flow Systems, San Diego, CA, USA).

### aCGH

DNAs were extracted using QIAGEN micro kits (Valencia, CA, USA). For each hybridization, 100 ng of genomic DNA from each sample and of pooled commercial 46XX reference (Promega, Madison, WI, USA were amplified using the GenomiPhi amplification kit (GE Healthcare, Piscataway, NJ, USA). Subsequently, 1 μg of amplified sample and 1 μg of amplified reference template were digested with DNaseI then labeled with Cy-5 dUTP and Cy-3 dUTP, respectively, using a BioPrime labeling kit (Invitrogen, Carlsbad, CA, USA). All labeling reactions were assessed using a Nanodrop assay (Nanodrop, Wilmington, DE, USA) prior to mixing and hybridization to CGH arrays with either 244,000 or 400,000 oligonucleotide features (Agilent Technologies, Santa Clara, CA, USA). The aCGH data have been deposited in the National Center for Biotechnology Information (NCBI) Gene Expression Omnibus (accession numbers GSE54328 and GSE21660).

### Resequencing

For each sequencing reaction 50 ng of DNA was amplified in 10 μl reactions for 35 cycles using MyTaq™ HSMix (Bioline, Taunton, MA, USA). All PCR products were verified by visual inspection on agarose gels. Samples were then purified by column filtration prior to analysis with an Applied Biosystems 3730 capillary sequencer (Life Technologies, Carlsbad, CA, USA). We surveyed 33 of the 35 exons in *STAG2* using published primers [3]. All sequences were analyzed using Mutation Surveyor v4.0.5 (Softgenetics, State College, PA, USA). *KRAS* mutational status was determined using custom primers designed to flank, amplify and sequence codons 12, 13 and 61 in exons 2 and 3 of the *KRAS* gene (Caris MPI, Irving, TX, USA) [17]. The *STAG2* resequencing data have been deposited in the NCBI BankIt (ID 1699484).

### Clinical patient samples, immunohistochemistry, and tissue microarray analysis

Tissue microarrays of 459 patients with 344 patients eligible for analysis were prepared from patient samples obtained after appropriate informed consent in Dresden (Institute of Pathology, University Hospital Dresden), Regensburg (Institute of Pathology, University Hospital Regensburg) and Jena (Institute of Pathology, University Hospital Jena). All samples were obtained with approved consent of the ethics committee of the Technischen Universität Dresden. Samples were collected consecutively from patients undergoing routine surgery for pancreatic carcinoma. Histological diagnosis was performed in the individual centers by pathologists trained in the routine work-up of pancreatic cancer specimens. The tumor samples were collected from 1993 to 2010, and most of the patients (68%) did not undergo adjuvant chemotherapy. Those that did undergo adjuvant therapy (32%) were chiefly treated with 5-fluorouracil (5-FU) or gemcitabine-based regimens and received a survival benefit of median 5.5 months (*P* = 0.02). The median survival times of patients after surgery from each center were indistinguishable. Immunohistochemistry was performed on 5 μm sections that were prepared using silanized slides (Menzel Gläser, Braunschweig, Lower Saxony, Germany). Staining was performed manually. In brief, sections were treated in PTM buffer pH6.0 for 45 minutes in the pressure cooker. After blocking of the endogenous peroxidase, the sections were incubated with the STAG2 antibody sc-81852 (1:400 for 30 minutes at room temperature). Antibody binding was detected using the Ultravision LP detection System (Thermo Fisher Scientific, Fremont, CA, USA) and Bright DAB (Medac, Wedel, Germany). Slides were counterstained with hematoxylin. Staining of STAG2 immunohistochemistry was performed by one (CP) and checked by another scientist (DA) with random point controls. Staining intensities were scored as the percentage of stained nuclei independent of staining intensity. Samples with a minimal staining of less than 30% on nuclei were scored as negative.

### Insertional mutagenesis screen

The generation and characterization of the KCTSB13 cohort, and the common insertion sites (CISs) analysis is described in [9].

### Detection of STAG2-T2/Onc fusion mRNA by RT-PCR in Sleeping Beauty tumors

Total RNA was extracted from snap-frozen pancreatic tumors developed in KCTSB13 mice using the RNeasy Mini kit (QIAGEN), and total RNA (1 μg) was reverse transcribed into cDNA using the High Capacity RNA-to-cDNA kit (Applied Biosystems). RT-PCR was carried out with a nested PCR approach using primers of mouse *STAG2* exon 1 and the carp β-actin splice acceptor sequence of the T2/Onc transposon cassette. cDNA was used as a template in a first round of PCR using specific primers corresponding to exon 1 of *STAG2* (5′-GAGGGAACAACATTCATGTG-3′) and the carp β-actin splice acceptor sequence (5′-CATACCGGCTACGTTGCTAA-3′). The product of this reaction was used as a template in a second round of nested PCR using an internal primer in the *STAG2* exon 1 (5′-CCCTCGGCTTCTCTCCCCCG-3′) and a second primer in the carp β-actin splice acceptor sequence (5′-ACGTTGCTAACAACCAGTGC-3′). PCR products were cloned into pCR 2.1-TOPO vector (Invitrogen) and positives clones sequenced.

### Cell culture

The 13 pancreatic and 3 control cell lines in this study were obtained directly from ATCC, which performs cell line characterizations using short tandem repeat profiling [18], and passaged for fewer than 6 months after resuscitation. Cells were maintained in RPMI medium (Invitrogen) supplemented with 10% fetal bovine serum (FBS) and penicillin/streptomycin (Invitrogen), and passaged for 3 months for all assays.

### Western blot analysis

The 13 PDA cell lines and 3 control cell lines (SK-ES-1, U87-MG, and A375) were lysed in buffer (Roche, Indianapolis, IN) containing protease and phosphatase inhibitors. Total protein (25 μg per sample) from each cell line was resolved on NuPAGE Novex 4-12% Bis-Tris precast gels (Invitrogen) then transferred to PVDF membranes. Antibodies against β-actin (Cell Signaling Technology, Danvers, MA, USA) and STAG2 (Santa Cruz Biotechnology, sc-81852, Santa Cruz, CA, USA) were used at 1:30,000 and 1:100 dilutions, respectively. Amersham ECL Prime Western Blotting Detection Reagent (GE Healthcare) was used to detect antibody binding on a BioSpectrum Imaging System (UVP, Upland, CA, USA).

### Small interfering RNA

The pancreatic cancer cell lines PANC-1 and Panc 04.03 were reverse transfected with four small interfering RNA (siRNA) sequences (QIAGEN) targeting *STAG2*, in addition to GFP, UBB, and ACDC control siRNAs (QIAGEN). siRNA (1 μl of 0.667 μM) was printed into each well of barcoded 384-well plates with a solid-white bottom (Corning 8749, Corning, NY, USA) using a Biomek FX Laboratory Automation Workstation (Beckman Coulter, Indianapolis, IN, USA). A transfection reagent, SilentFect lipid (Bio-Rad, Hercules, CA, USA), was used to introduce the siRNA sequences into the cells. A mixture of SilentFect and serum-free RPMI medium was added to the plates (20 μl per well) using a BioTek μFill Dispenser (BioTek, Winooski, VT, USA). Plates were then incubated for 30 minutes at room temperature to allow the formation of transfection reagent-nucleic acid complexes. Cells were trypsinized, quantified and resuspended in 10% FBS-RPMI assay medium and dispensed into the plates (20 μl per well) containing the siRNA using a μFill Dispenser (1,000 cells per well for PANC-1, 2,000 cells per well for Panc 04.03). Cells were incubated at 37°C for 24 hours before treatment with varying concentrations of each of 18 drugs (ranging from 0.6 nM to 100 μM) currently in use in our clinical trials, or vehicle alone in medium with 5% FBS by dispensing a 10 μl volume per well. The plates were further incubated at 37°C for 5 days before cellular viability was measured using CellTiter-Glo luminescent reagent (Promega) and an Analyst GT Multimode Microplate Reader (Molecular Devices, Sunnyvale, CA, USA). The final assay volume (per well) contained a 13 nM concentration of each siRNA, 40 nl of SilentFect transfection reagent and 5% FBS (20 μl serum-free medium + 20 μl of medium with 10% FBS + 10 μl of drug solution prepared in medium with 5% FBS).

## Results

**Figure 1:**
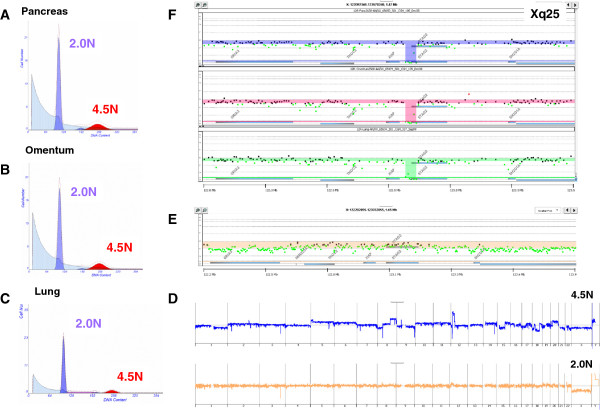
**Homozygous deletion of *STAG2 *in metastatic PDA.** A 4.5 N aneuploid population was detected and sorted in biopsies from the **(A)** primary and **(B,C)** two metastatic sites from rapid autopsy specimen UNMC 12R. **(D)** aCGH analysis of the sorted diploid (2.0 N; bottom) and the sorted 4.5 N (top) populations. Chromosome Xq25 CGH plots of **(E)** sorted 2.0 N population and **(F)** clonal homozygous deletion of *STAG2* in the 4.5 N populations sorted from the pancreas (top panel), omentum (middle panel), and lung (bottom panel). Shaded areas in (E,F) denote ADM2 step gram defined genomic intervals.

Rapid autopsy samples, consisting of patient-matched primary and distant metastatic tissues, have been used to study the clonal evolution of PDA [13,19]. In our studies we have screened multiple examples of these tumors using our flow sorting methods to identify clonal populations of PDA cells for genome analysis. In one of these cases we detected a 42.5 kb homozygous deletion in *STAG2* in a 4.5 N population sorted from the primary pancreatic tissue (Figure 1). The clonal deletion mapped to the 5′ region of the gene and included exon 1 and a series of regulatory regions [20]. Our use of flow sorted samples allowed objective discrimination of homozygous loss with a rigorous threshold of log_2_ratio < -3.0 in our aCGH results. The same homozygous deletion was detected in the 4.5 N aneuploid populations found in the two distinct distant metastatic sites surveyed within the same patient. In contrast, the patient-matched sorted diploid population had a normal aCGH profile with an intact *STAG2* locus. The presence of the same somatic homozygous deletion within a 4.5 N PDA population in each anatomical site suggests that cells that lost *STAG2* arose early in the history of this tumor and contributed to its progression to metastatic disease. Tumor suppressor genes targeted by homozygous deletions are frequently inactivated by alternative mechanisms, including somatic mutations [21,22]. Given the role of chromosomal instability and aneuploidy in the development and progression of PDA, we hypothesized that *STAG2* would be somatically deficient in a subset of PDA patients.

**Figure 2:**
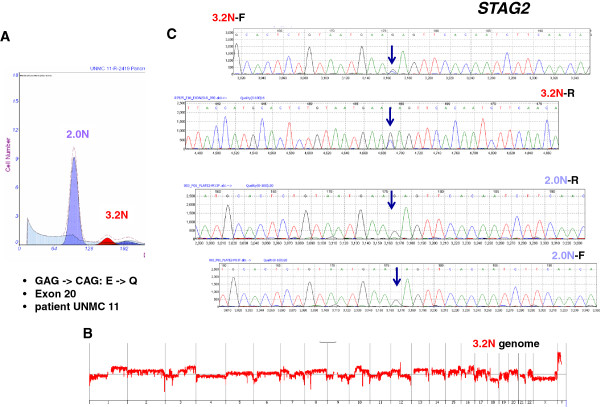
**Somatic mutation of *STAG2 *in metastatic PDA. (A)** Flow cytometry analysis and sorting of a 3.2 N aneuploid population in primary pancreas tissue from rapid autopsy specimen UNMC 11R. **(B)** Whole genome aCGH plot of the 3.2 N population. **(C)** Detection of somatic mutation in forward (3.2 N-F) and reverse (3.2 N-R) sequences in the 3.2 N genome. Mutation was not detected in the matching 2.0 N population sorted from the same tissue.

To identify somatic mutations we sequenced the first 33 of the 35 *STAG2* exons in 50 flow sorted clinical samples. Tissue samples were obtained from three sources. The first was a phase II trial of patients with advanced metastatic disease that had progressed on at least one prior therapy. The second was a phase III trial of patients with resectable PDA. The final source was a tumor bank that included tissues from a series of rapid autopsy samples. Whenever possible we used a patient matched blood sample as a control. However, for those samples of interest without matching normal tissue samples we evaluated the flow cytometry and aCGH profiles of the diploid and aneuploid fractions in each biopsy. In all cases the aneuploid fractions represent pure (>95%) tumor populations as determined by their separation from other peaks in the histograms and the presence of distinct genomic copy number aberrations, including homozygous deletions (log_2_ratios < -3.0) and focal amplicons. In contrast, the total diploid fractions from PDA biopsies may contain admixtures of neoplastic and non-neoplastic cells. Thus, for each sample of interest we profiled the total diploid fraction by aCGH. In all cases the genomes of the sorted diploid cells were non-aberrant by copy number analysis. This allowed discrimination of germ line from somatic events for tumor samples of interest, including those tissues without matching blood samples. A non-conserved *STAG2* mutation, E20Q, was found in one additional patient sample (Figure 2). The somatic nature of this mutation was confirmed by sequencing the 2.0 N population sorted from the primary tumor sample. These data provide the first report of *STAG2* somatic aberrations in human PDA. In addition we detected six recurring previously reported polymorphisms, and one novel polymorphism, throughout the gene in multiple patient samples (Additional file 1).

The previously reported transposon-mediated mutational screen of a pancreatic ductal preneoplasia mouse model identified a series of genes whose targeted inactivation cooperated with *KRAS*^G12D^ in the development and progression of PDA [9]. Strikingly, *STAG2* was identified as one of the top 20 candidate genes. Transposon insertions within the *STAG2* locus were found in 37/198 (18.7%) tumors that arose in the mouse model. In this present study we confirmed that *STAG2* expression was disrupted in these tumors by isolating chimeric fusion mRNAs that spliced the *STAG2* transcript to the T2/Onc transposon (Additional file 2). The insertions were found in preinvasive lesions and primary tumors, suggesting that early inactivation of the gene could be important in the progression of the disease. Insertions were also found in eight mice with metastasis. This included one animal with sufficient tumor tissue available for resequencing from primary and multiple metastases that confirmed the clonal nature of the *STAG2* insertion. Interestingly, the insertions are found in both males and females, suggesting that X-inactivation or haploinsufficiency could be contributing to the loss of *STAG2* expression in the females.

**Figure 3:**
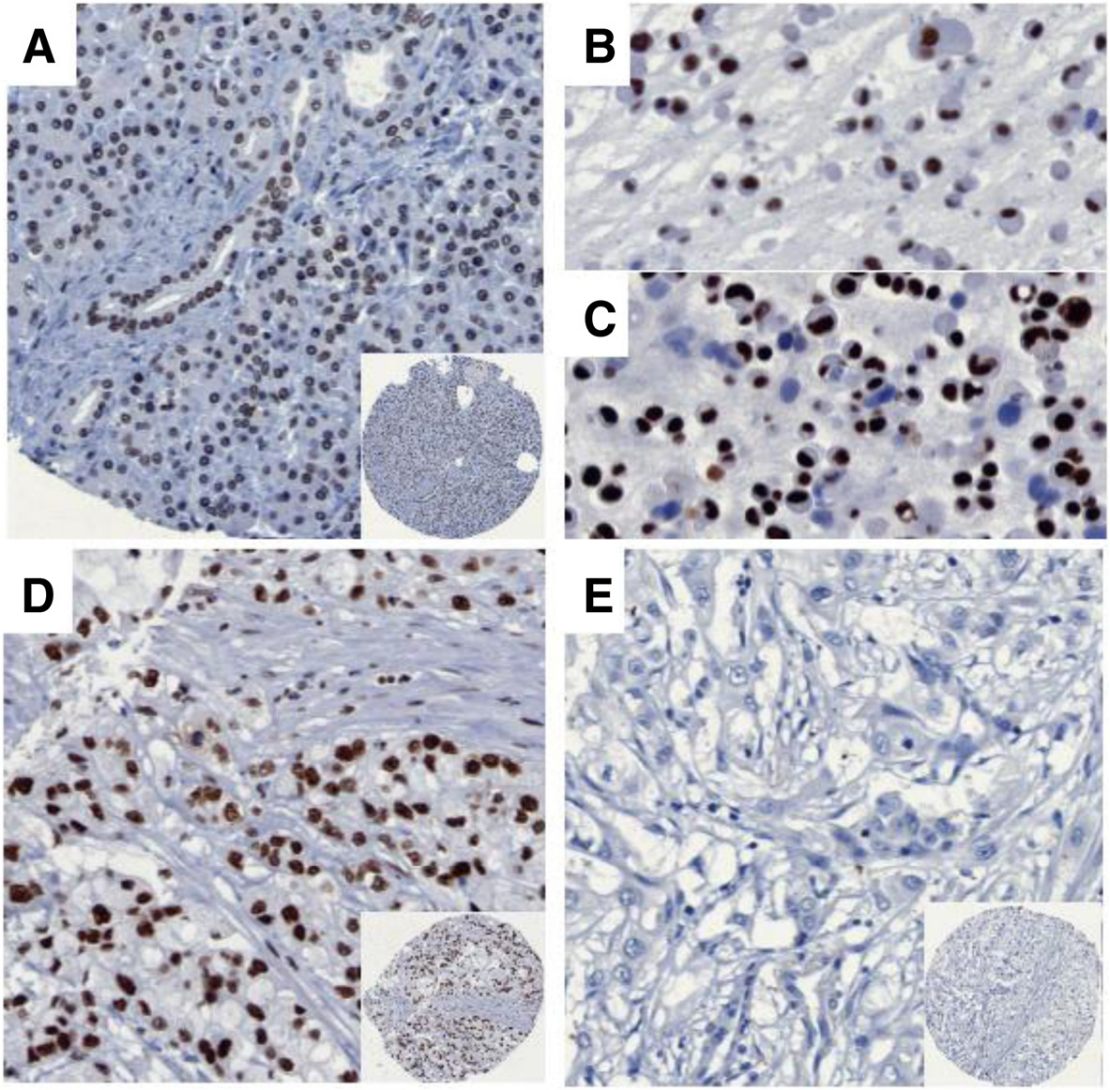
**Immunohistochemical staining of STAG2 in pancreatic cancer.** Near ubiquitous staining in **(A)** ductal cells of normal tissue, **(B)** the primary cancer cell line PaCaDD161, and **(D)** pancreatic cancer. **(C)** The cell line PaCaDD135 displays a heterogeneous staining pattern, whereas in **(E)** a minor fraction of pancreatic cancer STAG2 expression is lost. Magnification 200× (insets 100×).

**Figure 4:**
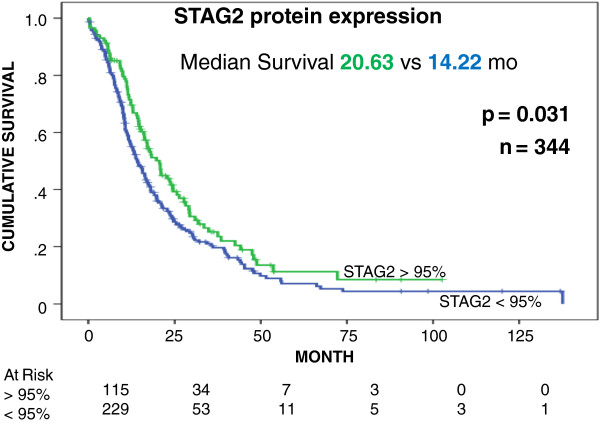
**STAG2 expression and overall survival in patients with PDA.** Tissue microarray (TMA) analysis of STAG2 protein expression in PDA samples from 344 patients. Cumulative survival (y-axis) of patients with intact versus deficient STAG2 expression levels is plotted versus time (x-axis). Mo, months.

**Figure 5:**
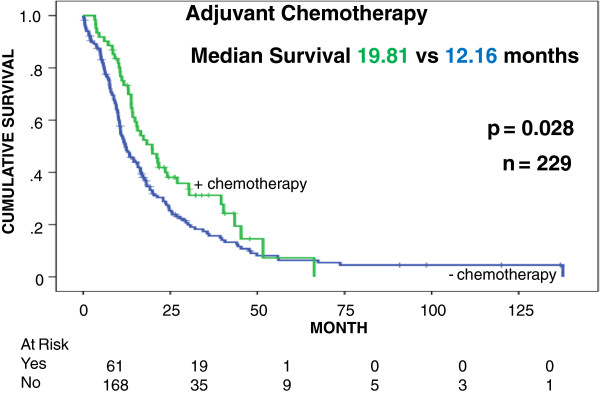
**STAG2 expression and response to adjuvant therapy.** Cumulative survival (y-axis) plotted versus time (x-axis) is compared for the 229 STAG2-deficient patients who received (n = 61) or did not receive (n = 168) adjuvant chemotherapy. Mo, months.

To further assess the clinical significance of STAG2 expression in human tumors, we screened a TMA containing a collection of 344 specimens obtained from resected German patients (Additional file 3). In normal tissue nearly all ductal cells stained with a high intensity (Figure 3). There was a broad range of signal intensities with a statistically significant loss of STAG2 expression in the tumor tissue (Wilcoxon rank test) and complete absence of STAG2 staining observed in 15 (4.3%) patients (Additional files 4 and 5). In univariate Kaplan-Meier analysis nearly complete STAG2-positive staining (>95% of nuclei positive) was associated with a median survival benefit of 6.41 months (*P* = 0.031) (Figure 4). Interestingly, the survival benefit of adjuvant chemotherapy can only be identified in the group of patients with a STAG2 staining <95% (median survival benefit 7.65 months; *P* = 0.028) (Figure 5). Multivariate cox regression analysis showed that STAG2 is an independent prognostic factor for survival in pancreatic cancer patients (Table 1).

**Table 1:** Multivariate analysis of tissue microarray cohort

**Model**	**Variable**	***P*-value**	**Hazard ratio**
			**(95% CI)**
A. Clinical pathology and STAG2	STAG2 (< 95%)	0.017	1.421 (1.064-1.898)
	T stage (1/2)	0.729	1.066 (0.743-1.529)
	N stage (N0)	0.196	0.826 (0.618-1.104)
	Grade (1/2)	0.013	0.711 (0.544-0.93)
	Sex (female)	0.865	1.023 (0.785-1.333)
	Margin involvement (negative)	0.297	0.852 (0.63-1.151)
	M stage (M0)	0.132	1.487 (0.887-2.492)
B. Clinical pathology and STAG2	STAG2 (< 95%)	0.017	1.415 (1.064-1.883)
	T stage (1/2)	0.719	1.068 (0.746-1.531)
	N stage (N0)	0.195	0.825 (0.618-1.103)
	Grade (1/2)	0.013	0.713 (0.546-0.931)
	Margin involvement (negative)	0.3	0.853 (0.631-1.152)
	M stage (M0)	0.126	1.494 (0.894-2.497)
C. Clinical pathology and STAG2	STAG2 (< 95%)	0.016	1.42 (1.068-1.888)
	N stage (N0)	0.198	0.827 (0.619-1.105)
	Grade (1/2)	0.014	0.717 (0.55-0.934)
	Margin involvement (negative)	0.321	0.861 (0.64-1.158)
	M stage (M0)	0.131	1.484 (0.889-2.478)
D. Clinical pathology and STAG2	STAG2 (< 95%)	0.014	1.428 (1.074-1.898)
	N stage (N0)	0.185	0.822 (0.616-1.098)
	Grade (1/2)	0.011	0.709 (0.545-0.924)
	M stage (M0)	0.086	1.556 (0.94-2.574)
E. Clinical pathology and STAG2 (final model)	STAG2 (< 95%)	0.021	1.394 (1.051-1.849)
	Grade (1/2)	0.007	0.697 (0.536-0.906)
	M stage (M0)	0.061	1.615 (0.979-2.665)

CI, confidence interval.

**Figure 6:**
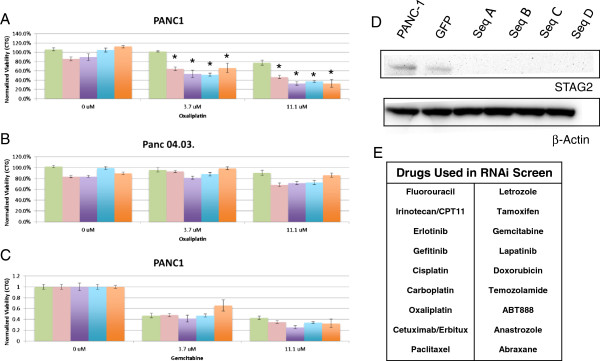
**Synthetic lethal analysis of STAG2 knockdown and exposure to chemotherapeutic agents in PDA cells. (A)** Synthetic lethal response of PANC-1 cells and **(B)** Panc 04.03 cells in the presence of oxaliplatin. **(C)** Absence of response of PANC-1 cells in the presence of gemcitabine. Asterisks indicate that all four siRNA sequences at drug concentrations of 3.7 and 11.1 μM have *P* < 0.01 when compared to GFP control. Error bars in (A-C) represent standard deviations. **(D)** Western blot of STAG2 expression in PANC1 cells in the presence of control (GFP) and four *STAG2* siRNAs. **(E)** Drugs used in siRNA synthetic lethal screen.

Given the role of STAG2 in the maintenance of genomic stability, we hypothesized that loss of STAG2 would create a synthetic lethal condition to exposure to one or more therapeutic agents. To test this we screened 13 PDA cell lines for expression of STAG2 by western blot analysis (Additional file 6). These included five cell lines previously reported as wild type [3]. All 13 cell lines have been shown to express *STAG2* mRNA; however, 2 of the cell lines had decreased protein levels relative to the other cell lines (Additional file 7) [23]. We selected a cell line (PANC-1) positive for STAG2 protein expression and one cell line (Panc 04.03) with low STAG2 protein expression for siRNA assays. Each of these two cell lines was exposed to 8 concentrations of the 18 clinical drugs available from our recently completed clinical trial in the presence or absence of 4 siRNAs targeting *STAG2* (Figure 6). Depletion of STAG2 did not affect the viability of these cell lines in the absence of drug. The STAG2-positive PANC-1 cell line displayed an increased sensitivity to the three platinum-based drugs (cisplatin, carboplatin, oxaliplatin) used in our assays in the presence of *STAG2* targeting siRNAs. For example, each of the four *STAG2* siRNAs significantly increased (*P* < 0.01) the sensitivity of PANC-1 cells to oxaliplatin at drug concentrations of 3.7, and 11.1 μM when compared to GFP control. In contrast, there was no difference in the response of the low STAG2 protein-expressing Panc 04.03 cells in the presence of any of the siRNAs. The knockdown of STAG2 in PANC-1 and Panc 04.03 cells was confirmed by western blot analysis for each of the siRNAs.

## Discussion

Somatic mutations in *STAG2* have now been reported in a variety of human cancers [3,8,24]. One notable exception to date has been pancreatic tumors. The initial analysis of *STAG2* in PDA was limited to five established cell lines [3]. These same cell lines were also positive for STAG2 protein expression in our current study (Additional file 6). In addition, recent next generation sequencing-based studies of the genomic landscape of PDA tumors have not detected somatic aberrations that selectively disrupted the *STAG2* locus [1,25]. Thus, to our knowledge, this report represents the first description of a tumor suppressor role for *STAG2* in human PDA. A key feature of our study of human tumors is the use of DNA content flow assays to identify and purify distinct populations of tumor cells in each biopsy of interest. These assays enable an unbiased analysis of clinical human PDA tissues regardless of tumor content. For example, in the two confirmed cases of somatic genomic targeting of *STAG2* the tumor cell content was less than 10% of the total cellular content of the biopsy (Figures 1 and 2). Our use of highly purified flow sorted tumor populations and the ability to detect homozygous deletions and mutations in PDA clinical samples, regardless of cellularity and tumor content, provides a robust screen for potential tumor suppressors.

Recent cancer genome studies have identified multiple examples of mutations and aberrations of potential driver genes in relatively small subsets (1 to 5%) of patients with a given tumor type [26-28]. Strikingly, many of these low frequency driver events occur in multiple tumor types, highlighting their potential clinical significance. Notably, a *STAG2* mutation was identified as a driver of clonal evolution from myeloid dysplastic syndrome to secondary acute myeloid leukemia [24]. The relatively low prevalence of *STAG2* somatic aberrations that we report in PDA is consistent with those in many other tumors [21]. The identification of *STAG2* as one of the most significant tumor suppressors of *KRAS*^G12D^-driven PDA in the GEM model supports an interactive role of these two genes in the development and progression of PDA. In our studies of human PDA we have confirmed the presence of *KRAS* mutations in over 95% of the flow sorted samples we have profiled. These include the two patients with *STAG2* somatic aberrations in the current study, one of whom had a clonal homozygous deletion in the primary tumor and two distant metastatic lesions. Thus, our current results further support the findings from the mouse model that *STAG2* inactivation cooperates with *KRAS* mutation as an early event in the evolution and progression of human PDA.

Our TMA-based expression analysis provides further clinical validation of a tumor suppressor role for *STAG2* in PDA. A recent study reporting *STAG2* mutations in bladder cancer included an immunohistochemistry screen across a broad panel of tumor types [8]. These included 36 PDA tumors, none of which showed loss of STAG2 expression. Tumor suppressor genes that are located on chromosome X are frequently subject to epigenetic regulation and silencing by chromosome X inactivation [29,30]. The reactivation of silent but intact copies of these tumor suppressors with agents such as 5-aza-2′-deoxycytidine and histone deacetylase (HDAC) inhibitors such as trichostatin has been proposed as a strategy to enhance therapeutic responses in solid tumors. The number of tumors (4.3%, 15/344) with a complete absence of protein expression in our panel of primary surgical resection tissues and our finding of somatic genetic lesions in 4% (2/50) of tumors surveyed reflects the overall prevalence of those PDAs with complete STAG2 loss (Additional file 8). However, a striking finding was the effect of even modest decreases of STAG2 protein levels on survival and response to adjuvant therapies (Figures 4 and 5). The presence of intact but under-expressed copies of this mediator of genome stability provides a potential therapeutic vulnerability similar to that proposed for the X-linked deubiquitinase *USP9X*[9]. Specifically, up-regulation of deficient but intact *STAG2* may provide a therapeutic benefit for patients with PDA, including those who have already progressed to invasive stages of disease. Furthermore, given the synergistic interaction with *KRAS* mutations, an early and ubiquitous genetic event in the development of PDA and the clinical significance of deficient expression, development of agents that increase STAG2 expression may provide a strategy for both treatment and prevention. However, a caveat for this approach is that, unlike *USP9X*, there is a low (approximately 4%) but potentially significant prevalence of genetic lesions in *STAG2* in human PDA.

Current therapeutic options for patients with advanced PDA include a series of chemotherapeutics with a broad range of mechanisms and targets (Figure 6). *STAG2* has been shown to regulate proper chromosome segregation in diploid cells. Loss of function gives rise to chromosome aneuploidy and a mutator phenotype in model systems. Thus, we hypothesized inactivation of *STAG2* could create a synthetic lethal combination with one or more currently used therapeutic agents that target either DNA replication or repair. Consistent with this, our TMA-based analysis of localized surgically resected tumors showed that those PDAs with deficient STAG2 levels derived the most benefit from standard adjuvant therapy with either gemcitabine or 5-FU. Our finding that RNAi-based silencing of *STAG2* sensitizes PDA cells specifically to platinum-based therapies *in vitro* suggests that the increased therapeutic benefit seen in patients receiving adjuvant therapies could be further enhanced. However, it remains to be determined if this added benefit may be limited to those relatively rare PDAs with complete loss of STAG2 expression.

## Conclusions

The clinical significance and translational importance of low frequency mutations and genomic lesions is a challenge to the study of PDA and other solid tumors. Our iterative approach, involving clonal genomic analysis of clinical samples, genetically engineered mouse models, clinical validation of genes of interest, and functional interrogation of candidate therapeutic targets and agents provides a comprehensive and highly translational approach to the study of PDA. This current work validates that *STAG2* behaves as a tumor suppressor gene in human PDA. Given its role in the maintenance of genome stability, the synergy with *KRAS* mutations, and the clinical significance of altered expression, we propose that deficiencies in *STAG2* represent a potential therapeutic vulnerability that can be exploited for improved treatment and possible prevention of PDA.

## Abbreviations

5-FU: 5-fluorouracil; aCGH: array comparative genomic hybridization; FBS: fetal bovine serum; GEM: genetically engineered mouse; GFP: green fluorescent protein; PCR: polymerase chain reaction; PDA: pancreatic ductal adenocarcinoma; RNAi: RNA interference; siRNA: small interfering RNA; TMA: tissue microarray.

## Competing interests

The authors declare that they have no competing interests.

## Authors’ contributions

MTB conceived the study. LE, TH, and EL did genomic characterization of human PDA samples. MA provided analysis of cell line expression data. NT and HY performed RNAi studies. MK did protein expression analysis of cell lines. The PDA mouse model studies were done by PP-M, and DAL. Construction, scoring, and analysis of TMA were done by DA, TK, PR, and CP. MTB, RR and DDVH reviewed all patient-based data. PP-M, CP, and MTB wrote the paper. All authors read and approved the final manuscript.

## Supplementary Material

Additional file 1: Table S1 *STAG2* SNPs detected in flow sorted PDA tissue samples. Click here for file

Additional file 2: Figure S1 Transposon insertion sites within the *STAG2* locus. *STAG2* was inactivated by insertional mutagenesis in a screen to identify genes that cooperate with K-Ras^G12D^ in the development of pancreatic cancer (Perez-Mancera *et al*. [9]). Isolation of the transposon insertion sites from 198 pancreatic tumor samples from the KCTSB13 cohort revealed a common insertion site in STAG2 in 18.6% of tumors, supporting its role as tumor suppressor gene in pancreatic cancer development. Transposon insertions parallel to *STAG2* expression are shown in green, while antiparallel insertions are shown in red. The lower panel shows the *STAG2* exon 1-T2/Onc chimeric mRNA in one of the tumors, confirming its inactivation by the transposon. Click here for file

Additional file 3: Table S2 Patient characteristics and TMA cohort. Clinical and histological parameters were used to calculate *P*-values with Kaplan-Meier statistics log-rank test. OS, overall survival. Daggers indicate the comparison of M0 versus M1 without the cases with Mx. Bold indicates *P*-values <0.05. Click here for file

Additional file 4: Figure S2 Distribution of STAG2 staining across 344 PDA samples. A histogram analysis of percentage STAG2 staining from TMA analysis in PDA patients. Click here for file

Additional file 5: Figure S3 Immunohistochemical staining of *STAG2* in pancreatic cancer. **(A-C)** Negative staining indicating loss of STAG2 expression in pancreatic cancers. The staining pattern is heterogeneous. **(D)** In a fraction of pancreatic cancer cells, however, STAG2 expression is lost and the surrounding stromal tissue displays no STAG2 staining. Magnification 130× in (A,B,D) (60×). Click here for file

Additional file 6: Figure S4 Western blot analysis of STAG2 expression in pancreatic cancer cell lines. Antibodies against β-actin (Cell Signaling Technology) and STAG2 (Santa Cruz Biotechnology, sc-81852) were used at 1:10,000 and 1:200 dilutions, respectively, against 20 μg of total protein from each of 13 pancreas and 3 control cell lines. Exposure times (60 minutes for STAG2 and 30 seconds for β-actin) were adjusted to account for the high and low molecular weights of the proteins and their differential signal intensities. Click here for file

Additional file 7: Figure S5 *STAG2* RNA expression in pancreatic cell lines. Box plot summary of gene expression RNA levels for 1,000 cell lines in Cancer Cell Line Encyclopedia (CCLE). The summary includes 44 pancreas cancer cell lines. Click here for file

Additional file 8: Figure S6 Survival time of STAG2-null (0%, n = 15) and STAG2-expressing (>0%, n = 329) PDA tumors. Kaplan-Meier curve of the 15 completely negative tumors versus the 329 tumors with varying degrees of positive staining. Click here for file
