# Supplementary material for: STAG2 is a clinically relevant tumor suppressor in pancreatic ductal adenocarcinoma
Source: Genome Med. 2014 Jan 31;6(1):9. doi: 10.1186/gm526 (PMC3971348; doi:10.1186/gm526)
Supplement: Additional file 1: Table S1 — STAG2 SNPs detected in flow sorted PDA tissue samples. [file gm526-S1.docx]

| **Supplemental Table 1** | | |
| --- | --- | --- |
| **Reference SNP Cluster Report (dbSNP)** | **MAF-1000 Genomes** | **Number of Patient Samples with SNP** |
| rs2297651 | 0.222/369 | 17 |
| rs34494691 | N/A | 2 |
| rs34397445 | .005/8 | 2 |
| rs200841330 | 0.370/614 | 26 |
| rs182757912 | 0.006/10 | 3 |
| rs200881211 | 0.002/3 | 2 |
