# Supplementary figures and images for: STAG2 is a clinically relevant tumor suppressor in pancreatic ductal adenocarcinoma
Source: Genome Med. 2014 Jan 31;6(1):9. doi: 10.1186/gm526 (PMC3971348; doi:10.1186/gm526)

## Slide 1
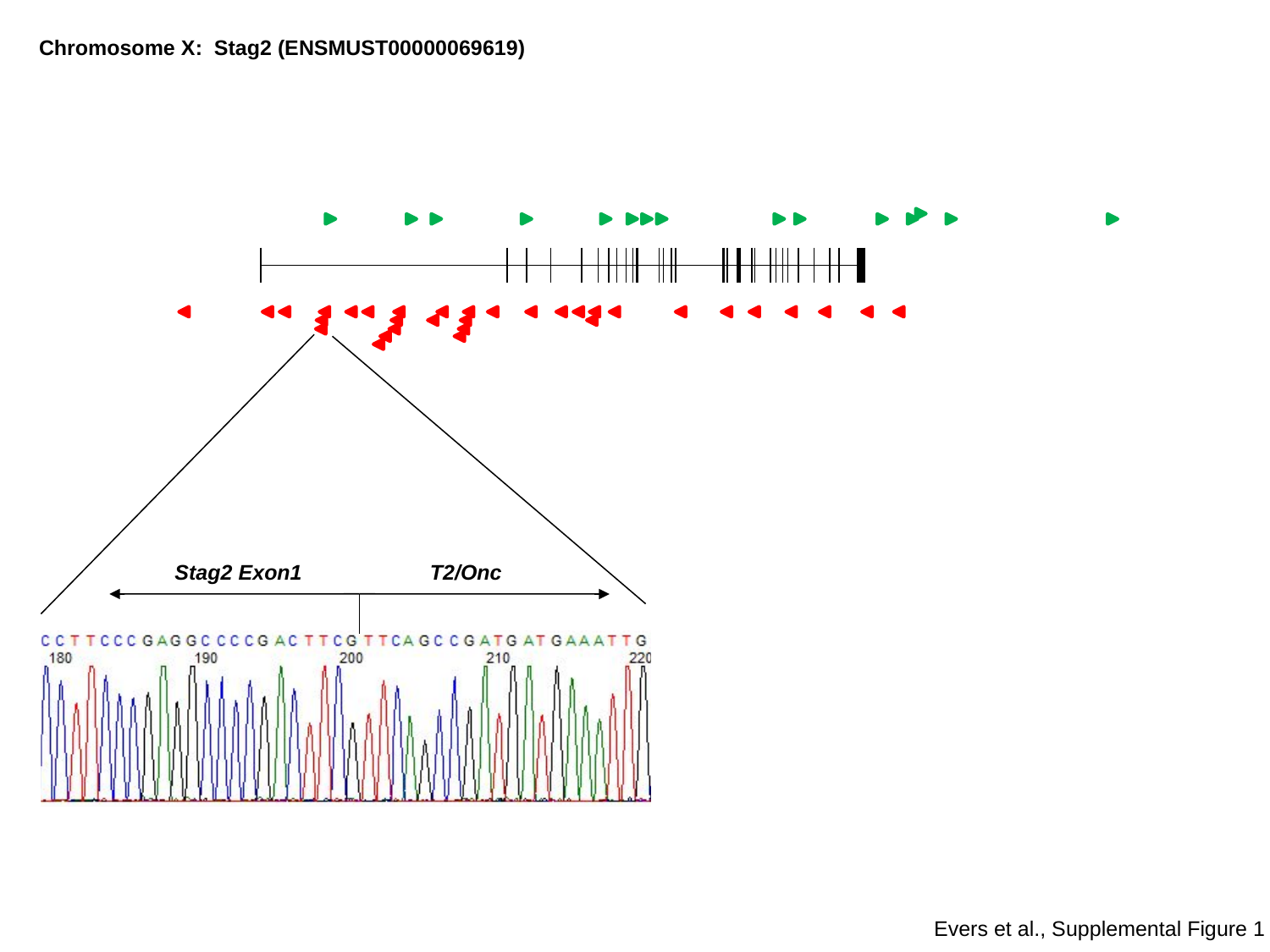

Chromosome X: Stag2 (ENSMUST00000069619)
Stag2 Exon1
T2/Onc
Evers et al., Supplemental Figure 1

Supplement: Additional file 2: Figure S1 — Transposon insertion sites within the STAG2 locus. STAG2 was inactivated by insertional mutagenesis in a screen to identify genes that cooperate with K-RasG12D in the development of pancreatic cancer (Perez-Mancera et al. [9]). Isolation of the transposon insertion sites from 198 pancreatic tumor samples from the KCTSB13 cohort revealed a common insertion site in STAG2 in 18.6% of tumors, supporting its role as tumor suppressor gene in pancreatic cancer development. Transposon insertions parallel to STAG2 expression are shown in green, while antiparallel insertions are shown in red. The lower panel shows the STAG2 exon 1-T2/Onc chimeric mRNA in one of the tumors, confirming its inactivation by the transposon. [file gm526-S2.pptx]

## Slide 1
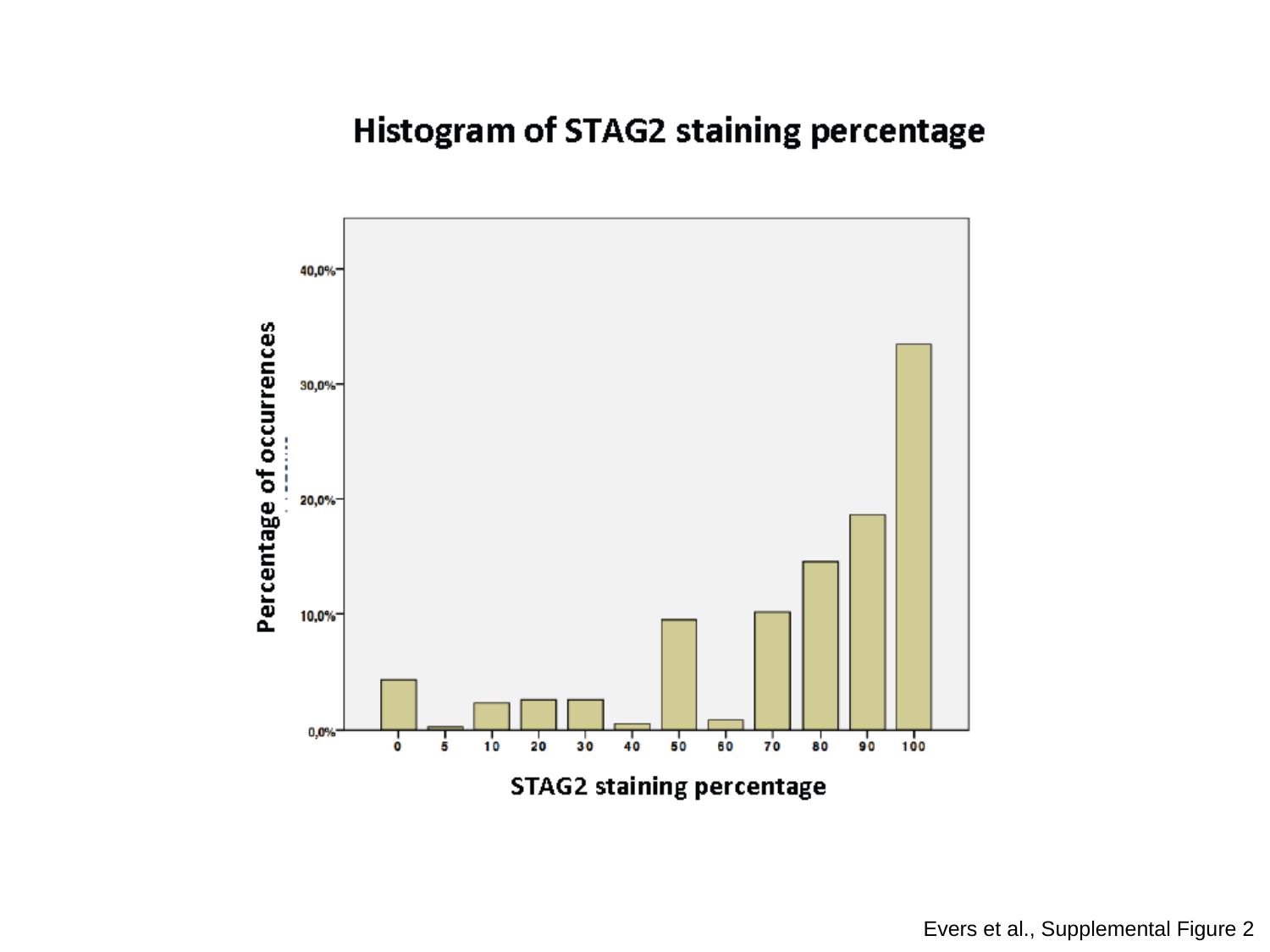

Evers et al., Supplemental Figure 2

Supplement: Additional file 4: Figure S2 — Distribution of STAG2 staining across 344 PDA samples. A histogram analysis of percentage STAG2 staining from TMA analysis in PDA patients. [file gm526-S4.pptx]

## Slide 1
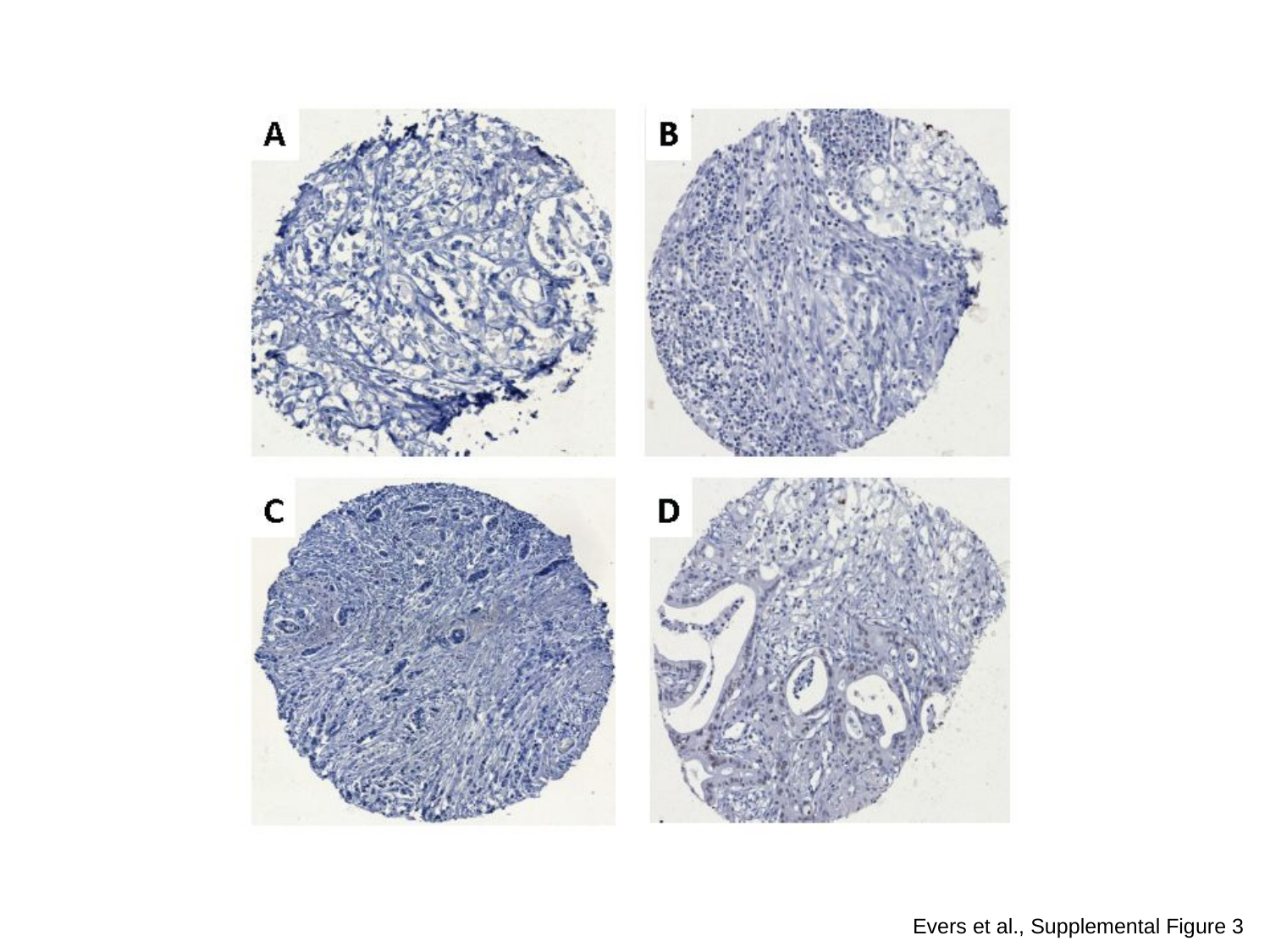

Evers et al., Supplemental Figure 3

Supplement: Additional file 5: Figure S3 — Immunohistochemical staining of STAG2 in pancreatic cancer. (A-C) Negative staining indicating loss of STAG2 expression in pancreatic cancers. The staining pattern is heterogeneous. (D) In a fraction of pancreatic cancer cells, however, STAG2 expression is lost and the surrounding stromal tissue displays no STAG2 staining. Magnification 130× in (A,B,D) (60×). [file gm526-S5.pptx]

## Slide 1
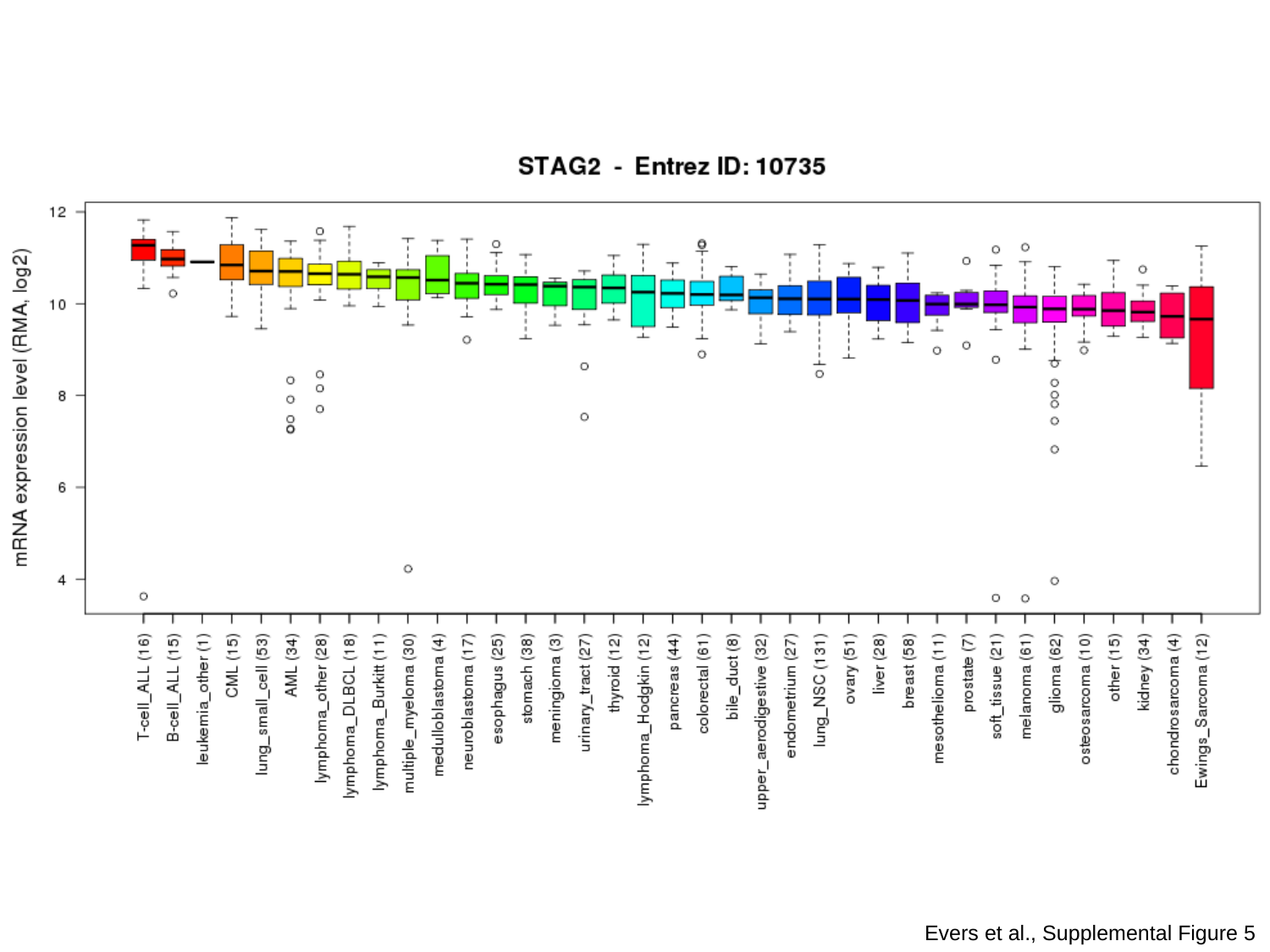

Evers et al., Supplemental Figure 5

Supplement: Additional file 7: Figure S5 — STAG2 RNA expression in pancreatic cell lines. Box plot summary of gene expression RNA levels for 1,000 cell lines in Cancer Cell Line Encyclopedia (CCLE). The summary includes 44 pancreas cancer cell lines. [file gm526-S7.pptx]

## Slide 1
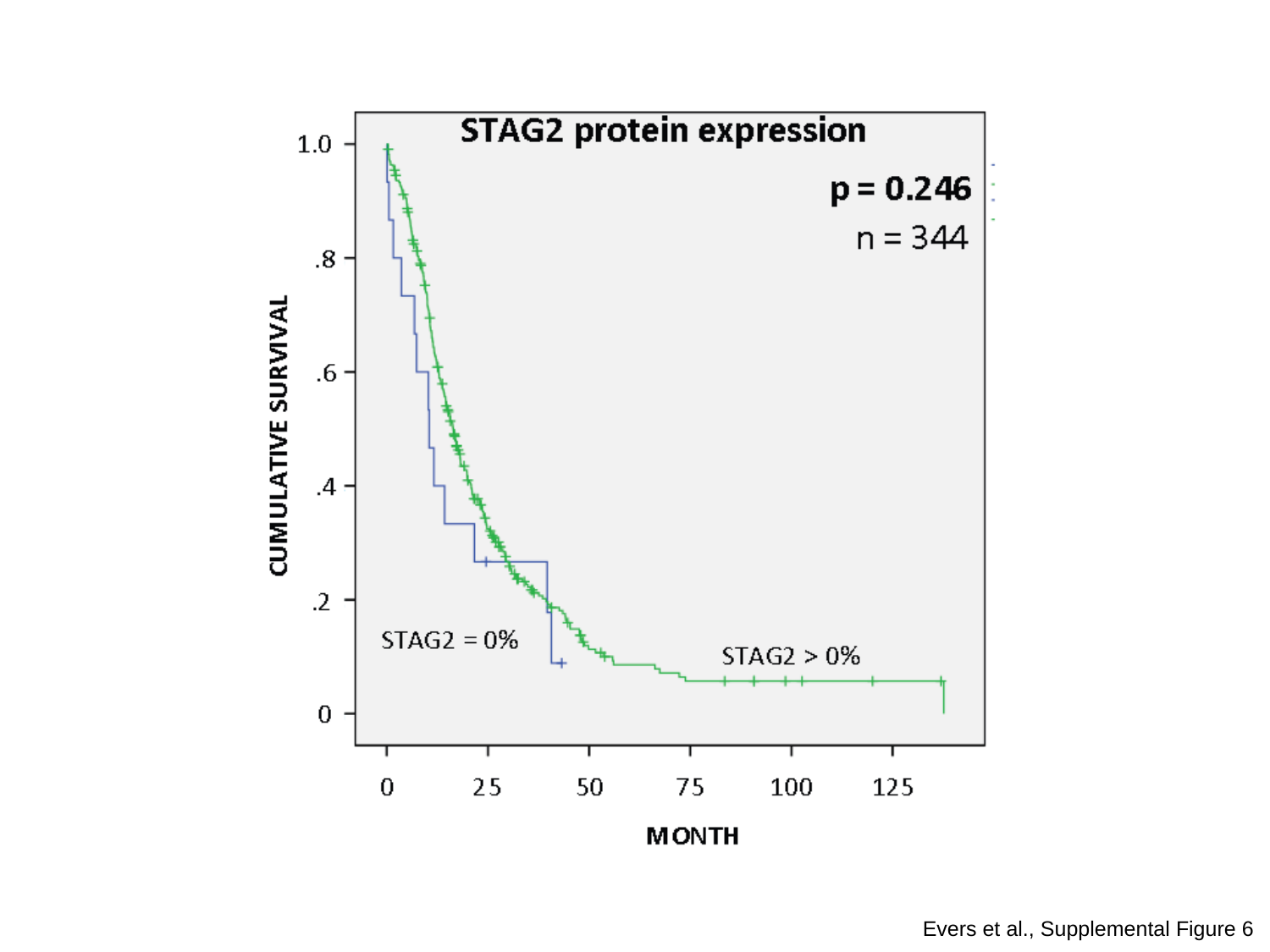

Evers et al., Supplemental Figure 6

Supplement: Additional file 8: Figure S6 — Survival time of STAG2-null (0%, n = 15) and STAG2-expressing (>0%, n = 329) PDA tumors. Kaplan-Meier curve of the 15 completely negative tumors versus the 329 tumors with varying degrees of positive staining. [file gm526-S8.pptx]
