# Supplementary material for: STAG2 is a clinically relevant tumor suppressor in pancreatic ductal adenocarcinoma
Source: Genome Med. 2014 Jan 31;6(1):9. doi: 10.1186/gm526 (PMC3971348; doi:10.1186/gm526)
Supplement: Additional file 3: Table S2 — Patient characteristics and TMA cohort. Clinical and histological parameters were used to calculate P-values with Kaplan-Meier statistics log-rank test. OS, overall survival. Daggers indicate the comparison of M0 versus M1 without the cases with Mx. Bold indicates P-values <0.05. [file gm526-S3.docx]

| **Supplemental Table 2** |
| --- |

| **Variables** | **N = 344 (%)** | **Median OS (mo)** | **P-value (Logrank)** |
| --- | --- | --- | --- |
| **Sex** |  |  |  |
| Male | 186 (54.1) | 16.87 |  |
| Female | 158 (45.9) | 14.52 | 0.703 |
| **Age (years)** |  |  |  |
| Mean | 64.03 |  |  |
| Median | 66.0 |  |  |
| Range | 33.0-84.0 |  |  |
| **Origin** |  |  |  |
| Dresden | 180 (52.3) | 16.29 |  |
| Jena | 93 (27.0) | 17.84 |  |
| Regensburg | 71 (20.7) | 13.76 | 0.897 |
| **T-Stage** |  |  |  |
| T1/2 | 46 (13.4) | 21.03 |  |
| T3/4 | 298 (86.6) | 15.34 | 0.659 |
| **N-Stage** |  |  |  |
| N0 | 106 (30.8) | 18.36 |  |
| N1 | 238 (69.2) | 15.60 | 0.069 |
| **M-Stage (interaortocaval lymph nodes)** | | | |
| M0 | 282 (82.0) | 16.79 |  |
| M1 | 18 (5.2) | 10.81 | 0.099† |
| Mx | 44 (12.8) |  |  |
| **Grade** |  |  |  |
| G1/2 | 171 (49.7) | 19.86 |  |
| G3/4 | 173 (50.3) | 14.29 | **0.003** |
| **Margins** |  |  |  |
| Clear | 258 (75.0) | 17.84 |  |
| Involved | 86 (25.0) | 12.88 | 0.058 |
| **Adj Chemotherapy** | |  |  |
| Yes | 110 (32.0) | 14.29 |  |
| No | 234 (68.0) | 19.81 | **0.020** |
| **STAG2 Expression** | |  |  |
| < 95% | 229 (66.6) | 14.22 |  |
| > 95% | 115 (33.4) | 20.63 | **0.031** |
