# Supplementary material for: STAG2 is a clinically relevant tumor suppressor in pancreatic ductal adenocarcinoma
Source: Genome Med. 2014 Jan 31;6(1):9. doi: 10.1186/gm526 (PMC3971348; doi:10.1186/gm526)
Supplement: Additional file 6: Figure S4 — Western blot analysis of STAG2 expression in pancreatic cancer cell lines. Antibodies against β-actin (Cell Signaling Technology) and STAG2 (Santa Cruz Biotechnology, sc-81852) were used at 1:10,000 and 1:200 dilutions, respectively, against 20 μg of total protein from each of 13 pancreas and 3 control cell lines. Exposure times (60 minutes for STAG2 and 30 seconds for β-actin) were adjusted to account for the high and low molecular weights of the proteins and their differential signal intensities. [file gm526-S6.pptx]

## Slide 1
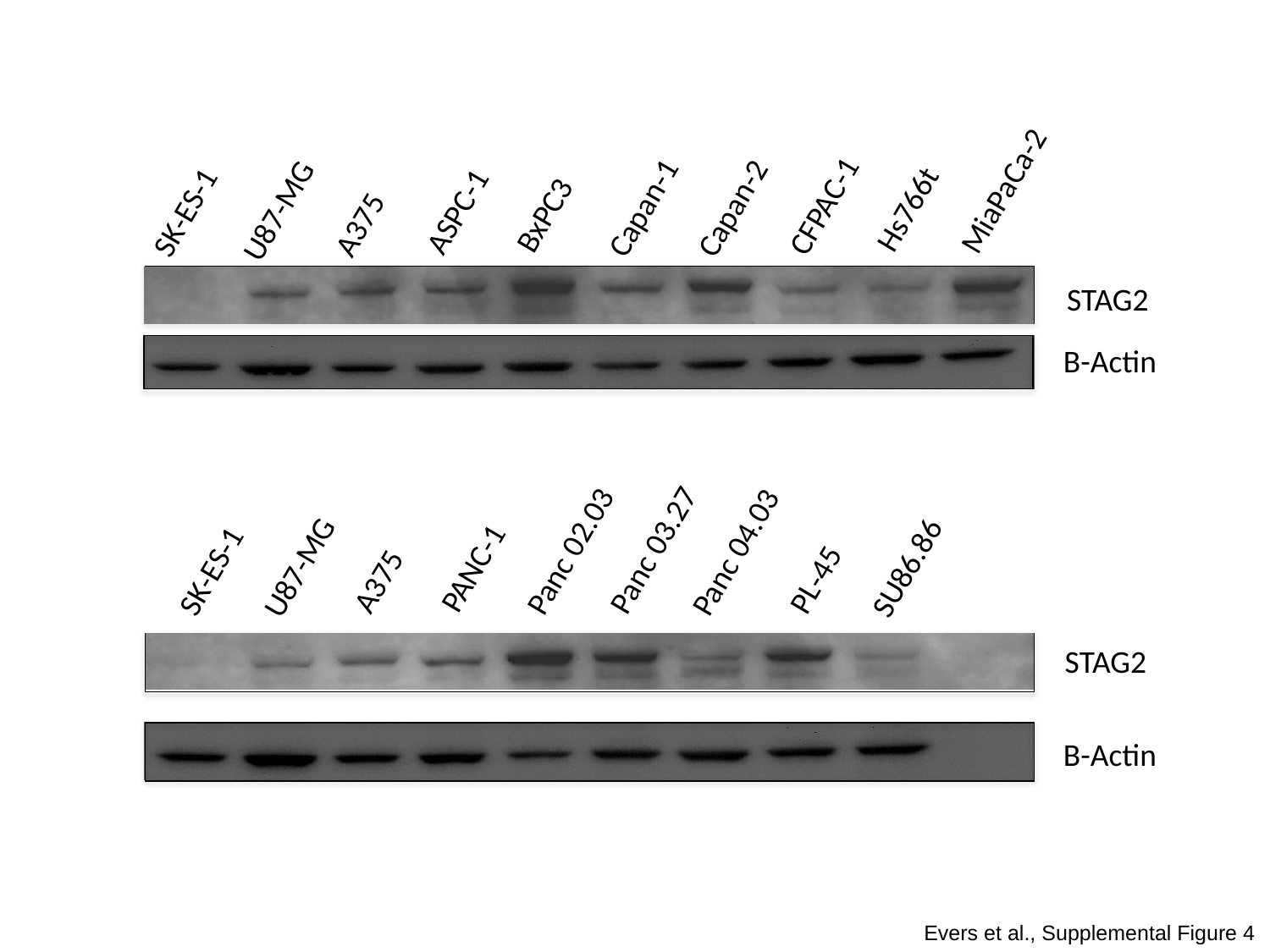

MiaPaCa-2
CFPAC-1
Capan-1
Capan-2
Hs766t
U87-MG
ASPC-1
SK-ES-1
BxPC3
A375
STAG2
Β-Actin
Panc 03.27
Panc 02.03
Panc 04.03
U87-MG
SU86.86
PANC-1
SK-ES-1
PL-45
A375
STAG2
Β-Actin
Evers et al., Supplemental Figure 4
